# Structural Features of a Dancong Tea Polysaccharide and Its Amelioration of Glucolipid Metabolic Disorders Associated with Gut Microbiota Modulation

**DOI:** 10.3390/foods15193364

**Published:** 2026-09-22

**Authors:** Run Lin, Zhe Wang, Zhiyang Chen, Ruisen Cen, Junqiao Wang, Xiaoyong Wu

**Affiliations:** 1 School of Food Science, Guangdong Pharmaceutical University, Zhongshan 528453, China; lin_run@outlook.com (R.L.);; 2 State Key Laboratory of Food Science and Resources, China-Canada Joint Lab of Food Science and Technology, Key Laboratory of Bioactive Polysaccharides of Jiangxi Province, Nanchang University, Nanchang 330047, China; 3 Guangdong Engineering Technology Research Center of Medicine-Food Homologous Foods, Guangdong Pharmaceutical University, Zhongshan 528453, China

**Keywords:** Dancong tea, polysaccharide, glucolipid metabolism, gut microbiota

## Abstract

A novel acidic polysaccharide, Dancong tea polysaccharide (DTP), was obtained from Dancong tea, and its structural characteristics and regulatory effects on glucolipid metabolism were investigated. With a molecular weight of 52 kDa, DTP is a pectic polysaccharide consisting mainly of a linear α-1,4-linked homogalacturonan backbone (72.7% galacturonic acid) with a methyl-esterification degree of 34.12%. In C57BL/6J mice fed a high-fat diet (HFD), administration of DTP effectively alleviated obesity, insulin resistance, dyslipidemia, and hepatic steatosis, which was accompanied by attenuated oxidative stress and inflammation. 16S rRNA gene sequencing demonstrated that DTP ameliorated gut dysbiosis through targeted regulation of specific taxa rather than a broad reversal at the phylum level. The medium dose of DTP reduced the abundance of obesity-associated genera (*Allobaculum*, *Dubosiella*), while high-dose DTP enriched beneficial *Bifidobacterium* species. Correlation analysis indicated that these microbial shifts were closely associated with the restoration of glucose and lipid homeostasis. Overall, our findings support the potential of DTP as a functional prebiotic ingredient for the management of metabolic diseases.

## 1. Introduction

With global economic development and lifestyle changes, the incidence of metabolic diseases, such as obesity, type 2 diabetes mellitus (T2DM), non-alcoholic fatty liver disease (NAFLD), and cardiovascular diseases, has increased markedly worldwide [1,2,3], posing a serious threat to public health. Glucolipid metabolic disorders, which are typically manifested as insulin resistance, hyperglycemia, dyslipidemia, and ectopic fat deposition, represent the central pathological basis driving the development of these diseases [4,5]. Accumulating epidemiological and experimental evidence has identified the Western-style diet, especially long-term intake of a high-fat diet (HFD) with high energy density and low fiber content, as a key environmental trigger of glucolipid metabolic disorders. In addition to causing excessive energy intake and fat accumulation directly, an HFD also disturbs systemic metabolic homeostasis by inducing insulin resistance, lipotoxicity, oxidative stress, and chronic low-grade inflammation [6,7,8,9,10]. Therefore, the development of safe and effective dietary intervention strategies against HFD-induced metabolic disorders is of great practical significance.

The gut microbiota, which is also known as the “second genome” of the human body, is deeply involved in nutrient metabolism, immune regulation, and the maintenance of host homeostasis [11,12]. Accumulating studies have demonstrated a close association between gut dysbiosis and HFD-induced glucolipid metabolic disorders [13]. Long-term HFD consumption markedly reshapes the gut microbial community, typically manifested as an increased Firmicutes/Bacteroidota (F/B) ratio, reduced microbial diversity, and an imbalance between beneficial bacteria (e.g., *Bifidobacterium*) and pathogenic bacteria [14,15,16]. Gut dysbiosis can compromise the intestinal barrier function, increase the translocation of endotoxins like lipopolysaccharide (LPS) into the bloodstream, a condition known as metabolic endotoxemia, thereby triggering chronic low-grade inflammation, which subsequently aggravates insulin resistance and lipid accumulation [17,18]. Notably, as important prebiotics, dietary polysaccharides can be selectively utilized by gut microbes to reshape the microbial community structure [19]. Accordingly, modulating the gut microbiota has become a promising strategy for the intervention of metabolic diseases.

Natural products are considered promising candidates for the prevention and adjuvant treatment of metabolic diseases owing to their abundant sources, multi-target actions, and relatively low toxicity [20]. Tea is one of the most widely consumed beverages worldwide and contains diverse bioactive constituents, such as polyphenols, alkaloids, amino acids, and polysaccharides [20,21]. Among these constituents, tea polysaccharides have attracted increasing attention because of their unique physicochemical properties and broad biological activities [22]. Polysaccharides derived from different teas, including black, green, and Pu-erh tea, have been reported to exhibit antioxidant, immunomodulatory, hypoglycemic, and hypolipidemic activities [23,24,25,26,27,28]. For instance, Pu-erh tea polysaccharides have been shown to ameliorate oxidative stress in diabetic mice by enhancing antioxidant enzyme activity [26]. Black tea polysaccharides can improve insulin resistance in T2DM mice via the PI3K/Akt pathway [27]. In addition, an acidic polysaccharide isolated from Wuyi rock tea was reported to produce hypoglycemic and hypolipidemic effects via regulating gut microbiota and bile acid metabolism [25]. Nevertheless, the bioactivities of tea polysaccharides are highly structure-dependent and vary with tea variety, geographical origin, and processing technology [22]. Dancong tea is a unique oolong tea variety from Guangdong, China, which is famous for its unique aroma and flavor, whereas its polysaccharide fractions have rarely been investigated [29]. In particular, the structural features of Dancong tea polysaccharides and their roles in the regulation of glucolipid metabolism remain largely unknown.

On this basis, a polysaccharide fraction (DTP) was isolated and purified from Dancong tea in the present study, and its structural characteristics were systematically characterized. Furthermore, the ameliorative effects of DTP on glucolipid metabolic disorders and its influence on the gut microbiota composition were evaluated in HFD-induced obese mice. The results of this study are expected to provide a scientific basis for the development of DTP as a functional food ingredient for the adjuvant management of metabolic diseases.

## 2. Materials and Methods

### 2.1. Materials and Reagents

Dancong tea (Milanxiang aroma type) was collected from Chaozhou, Guangdong Province, China, and processed as described by Hong et al. [30]. Dextran standards (T-10, T-25, T-40, T-50, T-70, T-500, and T-2000) were supplied by Shanghai Aladdin Biochemical Technology Co., Ltd. (Shanghai, China). Monosaccharide standards, including galacturonic acid, arabinose, galactose, rhamnose, glucose, fucose, fructose, ribose, and glucuronic acid, together with sodium borodeuteride and deuterium oxide (D2O, 99.9%), and methyl iodide were products of Sigma-Aldrich (St. Louis, MO, USA). Trifluoroacetic acid (TFA) and dimethyl sulfoxide (DMSO) were obtained from Aladdin Biochemical Technology Co. (Shanghai, China), while xylose and mannose were from J&K Scientific Co. (Beijing, China). Carbazole and the other analytical-grade reagents were provided by Macklin (Shanghai, China).

### 2.2. Preparation and Purification of DTP

The dried leaves of Dancong tea (250 g) were first refluxed with 5 L of 80% (*w*/*w*) ethanol at 75 °C for 60 min to eliminate small-molecule impurities such as lipids and pigments. The remaining residue was then extracted with 30 volumes of deionized water at 95 °C for 90 min. After filtration, the aqueous extract was concentrated under reduced pressure, and ethanol was added to a final concentration of 40% (*v*/*v*) to precipitate the polysaccharides. Following standing at 4 °C for 12 h, the precipitation procedure was repeated twice. The collected precipitate was redissolved in deionized water, decolorized on an NKA-9 macroporous resin column, and dialyzed (molecular weight cutoff of 8000–14,000 Da) against deionized water for 48 h. Finally, the retentate was lyophilized to afford the purified polysaccharide, which was designated DTP. The extraction procedure was independently repeated three times, and the overall yield of DTP was (1.10 ± 0.12)% of the dried tea leaves.

### 2.3. Sugar and Uronic Acid Content of DTP

The total sugar content of DTP was quantified by the phenol–sulfuric acid method using galactose as the calibration standard, while the uronic acid content was measured by the carbazole–sulfuric acid method using galacturonic acid as the calibration standard [31]. The protein content was determined by the Bradford method using bovine serum albumin as the calibration standard. The total sugar and uronic acid contents were calculated from the respective calibration curves and are expressed as mass percentages of the corresponding standard equivalents (galactose equivalents for total sugar and galacturonic acid equivalents for uronic acid) relative to the dry weight of DTP.

### 2.4. Structural Characterization of DTP

#### 2.4.1. Molecular Weight Determination

The homogeneity and molecular weight distribution of DTP were evaluated by high-performance gel permeation chromatography (HPGPC). DTP was dissolved in 0.1 mol/L NaCl at 1 mg/mL and passed through a 0.22 μm aqueous membrane before injection. A calibration curve was constructed with dextran standards and glucose for molecular weight calculation.

Chromatographic separation was carried out on a Waters Ultrahydrogel^TM^ Linear column (7.8 × 300 mm; Waters, Milford, MA, USA) connected to a differential refractive index detector (RID). The column was eluted with 0.1 mol/L NaCl at 0.6 mL/min and maintained at 35 °C, with an injection volume of 20 μL.

#### 2.4.2. Monosaccharide Composition Analysis

The monosaccharide composition of DTP was determined on a Dionex ICS 6000 high-performance anion exchange chromatography system equipped with pulsed amperometric detection (HPAEC-PAD; Thermo Fisher Scientific, Waltham, MA, USA). Considering the high uronic acid content of DTP, a two-step hydrolysis procedure involving methanolysis followed by sulfuric acid hydrolysis was adopted according to Hong et al. [30] to achieve complete cleavage of the glycosidic bonds. Briefly, DTP was first methanolyzed in 2 M HCl/methanol at 80 °C for 4 h and dried; the residue was then treated with 12 M H_2_SO_4_ for 30 min at room temperature under stirring, diluted to 2 M H_2_SO_4_, and further hydrolyzed at 100 °C for 3 h. The resulting hydrolysate was filtered (0.22 μm) and separated on a CarboPac™ PA20 analytical column (3 × 150 mm; Thermo Fisher Scientific, Waltham, MA, USA) using a gradient of water, NaOH, and sodium bicarbonate (NaHCO_3_) at 0.5 mL/min. Individual monosaccharides were quantified against the calibration curves of the corresponding standards. Before injection, the cooled hydrolysate was transferred to a 50 mL volumetric flask and brought to volume with deionized water, and a 2 mL aliquot was further diluted to 10 mL; the final sulfuric acid concentration in the injected hydrolysate was therefore 0.024 mol/L. The diluted hydrolysate was injected directly without neutralization or desalting, since the residual dilute sulfuric acid was neutralized in-line by the strongly alkaline eluent, which follows the established practice for HPAEC-PAD analysis of acid hydrolysates.

#### 2.4.3. Methylation and GC-MS Analysis

Glycosidic linkage analysis was conducted by methylation combined with GC-MS, following the protocol of Hong et al. [30] with substantial modifications. Because DTP is rich in galacturonic acid (GalA), a two-step carboxyl reduction was performed before methylation so that methyl-esterified and free uronic acids could be distinguished [32]. In brief, DTP dissolved in imidazole-HCl buffer was first treated with NaBD_4_ to reduce the methyl-esterified carboxyl groups; the remaining free carboxyl groups were then activated with carbodiimide and reduced with NaBH_4_. The carboxyl-reduced polysaccharide was methylated using a modified method [32]: the sample was dissolved in DMSO, and a NaOH-DMSO suspension together with methyl iodide (CH3I) was added to complete the methylation. The permethylated polysaccharide was hydrolyzed in 2 M TFA at 121 °C for 90 min, reduced with NaBD_4_ in 2 M ammonia, and acetylated with acetic anhydride to generate partially methylated alditol acetates (PMAAs). Analysis of the PMAAs was performed on an Agilent 7890B/7000D GC-MS system (Agilent Technologies, Santa Clara, CA, USA) fitted with an SP-2330 MS capillary column (30 m × 0.35 mm × 0.2 μm; Supelco, Bellefonte, PA, USA), with the oven temperature programmed from 160 °C to 210 °C at 2 °C/min and then to 240 °C at 5 °C/min (held for 20 min); mass spectra were recorded over *m*/*z* 35–400.

#### 2.4.4. Nuclear Magnetic Resonance (NMR) Analysis

NMR analysis was performed as described by Hong et al. [30]. To eliminate the interference of exchangeable protons from hydroxyl and carboxyl groups, DTP (approximately 30 mg) was exchanged with D2O (99.9%) and lyophilized, and this dissolution-lyophilization process was repeated three times. The deuterium-exchanged sample was then dissolved in 0.6 mL of deuterated DMSO (99.9%) for NMR measurement. Spectra were recorded at 25 °C on a Bruker Avance III HD 400 MHz spectrometer (Bruker, Zurich, Switzerland) equipped with a cryoprobe, including one-dimensional (1D) spectra (1^H^ and 13^C^) as well as two-dimensional (2D) correlation spectroscopy (COSY), total correlation spectroscopy (TOCSY), and heteronuclear single quantum coherence (HSQC) spectra. The 1H spectrum was acquired with the zg30 pulse program (128 scans, relaxation delay of 1 s, spectral width of 20.0 ppm, and 65,536 data points); the 13C spectrum with the zgpg30 pulse program (32,280 scans, relaxation delay of 2 s, spectral width of 238.9 ppm, and 65,536 data points); and the 2D spectra (COSY, TOCSY, and HSQC) were acquired with 128 scans per increment using the cosygpmfqf, mlevphpp, and hsqcetgp pulse programs, respectively, with relaxation delays of 2, 2, and 1.5 s and spectral widths of 18.2, 18.2, and 17.0 ppm in the 1H dimension and 220.8 ppm in the 13C dimension (HSQC), respectively.

### 2.5. Animal Experiments

Fifty male C57BL/6J mice (5 weeks old, SPF grade) were purchased from the Guangdong Medical Laboratory Animal Center (License No. SCXK(Guangdong)2022-0002). The mice were housed at 23 ± 2 °C under a 12 h light/dark cycle with ad libitum access to food and water. The standard chow diet (3.40 kcal/g, 11.85% energy from fat) was provided by the Laboratory Animal Center of Guangdong Pharmaceutical University, and the high-fat diet (HFD; 4.73 kcal/g, 45% energy from fat; Cat. No. GD45) was purchased from the Guangdong Medical Laboratory Animal Center. The HFD (formulated with reference to the D12451 diet) consisted of (g/kg): casein 200, L-cystine 3, corn starch 72.8, maltodextrin 100, sucrose 167.8, cellulose 50, soybean oil 25, lard 177.5, mineral mix S10026B 50, vitamin mix AIN-76A 10, and choline bitartrate 2, providing 24% protein, 41% carbohydrate, and 24% fat by weight (20%, 35%, and 45% of total energy, respectively). After one week of acclimatization, six mice were randomly assigned to the normal control group (Control) and fed the standard chow diet throughout the experiment, while the remaining mice were fed the HFD. After 2 weeks of HFD feeding, the 24 mice with the greatest body weight gain (top 60%) were selected and randomly divided into four groups (*n* = 6) using a random number table: a model group (Model) and three DTP-treated groups at low (DTP-L, 50 mg/kg·bw), medium (DTP-M, 100 mg/kg·bw), and high (DTP-H, 200 mg/kg·bw) doses. This selection criterion was prespecified prior to the experiment: since C57BL/6J mice exhibit heterogeneous responses to HFD feeding and a subset of the animals is relatively resistant to diet-induced obesity, selecting the mice with the greatest body weight gain is a commonly adopted strategy to ensure a uniform and stable obese phenotype and to reduce inter-individual variability before randomization; this approach follows the Health Food Function Inspection and Evaluation Methods (2023 Edition) for the body-fat-control function test, which prescribes eliminating the one-third of animals with the lowest body weight gain (obesity-resistant animals) and retaining the obesity-sensitive animals after the model-building period [33]; the remaining mice with lower body weight gain were excluded from the intervention experiment and were not subjected to any treatment or analysis. The sample size (*n* = 6 per group) was determined based on previous polysaccharide intervention studies using similar HFD-induced models [25,31], in which six mice per group were sufficient to detect significant differences in metabolic parameters. DTP was dissolved in physiological saline at concentrations of 5, 10, and 20 mg/mL for the DTP-L, DTP-M, and DTP-H groups, respectively, and was administered to the mice by oral gavage once daily at a volume of 0.1 mL/10 g body weight for 6 weeks; the Control and Model groups received an equal volume of physiological saline by gavage. All groups except the Control group were maintained on the HFD throughout the 8-week experimental period. Body weight and food intake were monitored daily.

At the end of the experiment, after a 12-h fast, fasting blood glucose (FBG) was measured. Mice were then anesthetized for blood collection via retro-orbital bleeding. Serum was prepared and stored at −80 °C. Subsequently, mice were euthanized, and key tissues (liver, epididymal, inguinal, subcutaneous white adipose tissue, and interscapular brown adipose tissue) and cecal contents were rapidly dissected, weighed, and snap-frozen for further analysis. All procedures were approved by the Animal Ethics Committee of Guangdong Pharmaceutical University (Ethics Approval No. SPF2022530).

### 2.6. Oral Glucose Tolerance Test (OGTT)

On day 42 of the experiment (one week before the end of the intervention), an OGTT was performed. This time point was chosen to evaluate glucose tolerance at a late stage of the intervention while avoiding the potential interference of the acute stress caused by fasting and repeated blood sampling with the terminal measurements and tissue collection at sacrifice. Briefly, mice were fasted for 12 h before being administered a glucose solution (2.0 g/kg body weight) via oral gavage. Blood was collected from the tail vein at 0, 30, 60, 90, and 120 min post-gavage to measure blood glucose levels. The area under the curve (AUC) for the OGTT was subsequently calculated.

### 2.7. Biochemical Parameter Determination

Serum total cholesterol (TC), triglycerides (TG), high-density lipoprotein cholesterol (HDL-C), and low-density lipoprotein cholesterol (LDL-C) were measured with commercial assay kits from Nanjing Jiancheng Bioengineering Institute (Nanjing, China; Cat. No. A111-1-1, A110-1-1, A112-1-1, and A113-1-1, respectively). Fasting insulin (FINS), leptin (LEP), and adiponectin (ADPN) in serum were quantified with ELISA kits from Quanzhou Ruisin Biotechnology Co. (Quanzhou, China; Cat. No. RX201610M, RX202629M, and RX202420M, respectively). Insulin resistance was evaluated by the homeostatic model assessment (HOMA-IR), calculated as FBG (mmol/L) × FINS (mU/L)/22.5 [34].

Hepatic TC and TG in liver homogenates were measured with commercial kits as well. Hepatic oxidative stress-related indices, including malondialdehyde (MDA), glutathione peroxidase (GSH-Px), catalase (CAT), and superoxide dismutase (SOD), were assayed with kits from Nanjing Jiancheng Bioengineering Institute (Cat. No. A111-1-1, A110-1-1, A003-1-2, A001-3-2, A005-1-2, and A007-1-1, respectively), and liver glycogen was determined with a kit from Quanzhou Ruisin Biotechnology Co. (Cat. No. RXWB0408). Inflammatory cytokines (IL-1β, IL-6, TNF-α, and IL-10) in liver tissue and serum were quantified with ELISA kits from Quanzhou Ruisin Biotechnology Co. (Cat. No. RX203063M, RX203049M, RX202412M, and RX203075M, respectively). Hepatic indices were all normalized to tissue total protein, which was measured with a BCA protein assay kit (Servicebio Technology Co., Wuhan, China; Cat. No. G2026).

### 2.8. Gut Microbiota Analysis

Total genomic DNA was extracted from cecal content samples stored at −80 °C using the E.Z.N.A.^®^ Soil DNA Kit (Omega Bio-tek, Norcross, GA, USA). The V3–V4 hypervariable region of the 16S rRNA gene was amplified from the extracted DNA using the universal primers 338F (5′-ACTCCTACGGGAGGCAGCAG-3′) and 806R (5′-GGACTACHVGGGTWTCTAAT-3′). Following purification and quantification, the PCR products were used to construct a sequencing library with the NEXTFLEX^®^ Rapid DNA-Seq Kit (Bioo Scientific, Austin, TX, USA). Sequencing was performed by Shanghai Majorbio Bio-pharm Technology Co., Ltd. (Shanghai, China) on an Illumina MiSeq platform using a MiSeq Reagent Kit v3 (Illumina, San Diego, CA, USA). Paired-end reads were merged based on their overlap relationship and quality-filtered. Operational taxonomic units (OTUs) were clustered at a 97% sequence similarity cutoff using UPARSE (v7.0.1090), and chimeric sequences were removed during clustering. The representative sequence of each OTU was taxonomically annotated against the SILVA 16S rRNA gene database (Release 138) using the RDP classifier (v2.11) with a confidence threshold of 0.7. Alpha diversity indices, including the Ace, Chao, Shannon, Simpson, and coverage indices, were calculated using Mothur (v1.30.2) based on the normalized number of reads. Beta diversity was evaluated by principal coordinates analysis (PCoA) and non-metric multidimensional scaling (NMDS) based on Bray–Curtis distances using QIIME (v1.9.1). Linear discriminant analysis effect size (LEfSe) analysis was performed to identify the differentially abundant taxa among groups (LDA score > 4.0, *p* < 0.05, all-against-all strategy). All bioinformatics analyses were conducted on the Majorbio Cloud Platform (https://cloud.majorbio.com).

### 2.9. Statistical Analysis

Data are presented as the mean ± standard error of the mean (SEM). All statistical analyses were performed with SPSS software (version 27.0). Differences among multiple groups were evaluated by one-way analysis of variance (ANOVA) combined with Tukey’s HSD post-hoc test. For non-parametric data, including the relative abundance of gut microbiota and alpha diversity indices, the Kruskal–Wallis H test was applied. For LEfSe analysis, the Kruskal–Wallis rank-sum test was first applied to detect features with significantly differential abundance among groups, and linear discriminant analysis (LDA) was then used to estimate the effect size of each differentially abundant feature. Associations between gut microbial taxa (at the phylum and genus levels) and host metabolic parameters were examined by Spearman’s rank correlation analysis, which covered the dominant taxa and key indicators, including body weight, serum lipids, blood glucose, and hepatic biochemical and inflammatory markers. Graphs were prepared with Origin 2022 and Adobe Illustrator 2023.

## 3. Results

### 3.1. Chemical Composition and Structural Characterization of DTP

DTP was prepared through hot-water extraction, 40% ethanol precipitation, and purification on an NKA-9 macroporous resin column. Chemical composition analysis showed that DTP was an acidic polysaccharide containing 40.24 ± 1.14% total sugar and 67.29 ± 4.34% uronic acid, together with a low protein content of 3.86 ± 0.33% as determined by the Bradford method. HPGPC analysis gave a molecular weight of approximately 52 kDa for DTP (Figure 1A). The small peaks eluting after the main peak in Figure 1A (approximately 20–23 min) are assigned to small molecules co-purified with DTP, which elute beyond the total permeation volume of the column (later than monosaccharides such as glucose) and are therefore attributed to non-sugar small molecules rather than saccharide components; notably, the smaller polysaccharide fraction present in the polysaccharide before dialysis was removed by the dialysis step (MWCO 8000–14,000 Da), as confirmed by the HPGPC chromatogram of the pre-dialysis sample (Figure S1). It should be noted that the moisture, ash, residual polyphenol, and caffeine contents of DTP were not determined in the present study and will be reported in our future work. It should also be noted that the total sugar and uronic acid contents were determined by two independent colorimetric assays with different standards (galactose equivalents and galacturonic acid equivalents, respectively); because the phenol–sulfuric acid assay exhibits a relatively low color response toward uronic acids whereas the carbazole–sulfuric acid assay is specific for uronic acids, the two values should not be simply summed.

Nine monosaccharides were detected in DTP (Table 1). According to the molar ratios of the major monosaccharides, the structural parameters of the pectic domains were estimated following reported methods [30,35]. The homogalacturonan (HG) and rhamnogalacturonan-I (RG-I) domains accounted for approximately 67.3% and 22.4% of DTP, respectively, and the R2 value (Rha/GalA) was below 0.1 (Table 1), suggesting that HG is the dominant structural domain of DTP, whereas RG-I represents only a minor fraction.

Methylation analysis identified no fewer than 20 glycosidic linkage types in DTP (Table 2), among which 1,4-linked GalpA and 1,4-linked GalpAOMe were predominant, together representing 71.57% of the total linkages and verifying that an α-1,4-linked HG chain constitutes the backbone of DTP. The degree of methyl-esterification (DM), estimated from the peak areas of methyl-esterified GalA relative to total GalA residues, was about 34.12%, and the degree of branching (DB) was 0.17, suggesting that DTP is a sparsely branched polysaccharide. Only 0.77% of 1,2-linked Rhap was detected. The RG-I proportion estimated from the monosaccharide ratios (22.4%) appears higher than that expected from the linkage data, which may be attributed to free neutral sugars or to the sensitivity of the calculation method to side-chain variations.

The structure of DTP was further resolved by 1D and 2D NMR spectroscopy. Although the ^1^H spectrum (Figure 1B) exhibited broad overlapping signals, several diagnostic resonances could be assigned: the intense signal at 3.67 ppm corresponded to the methyl ester protons (-OCH_3_) of GalA residues, and the major anomeric signal at 4.84 ppm (within the 4.5–5.5 ppm region) was typical of α-linked GalA. In the ^13^C spectrum (Figure 1C), the HG backbone gave rise to characteristic resonances at 100.36 ppm (anomeric carbon), 170.60 ppm (carboxyl carbon), and 52.74 ppm (methyl ester carbon).

The chemical shifts (1H and 13C) of the major sugar residues are summarized in Table 3, which shows that →4)-α-D-GalpA-6-OMe-(1→ (residue A) and →4)-α-D-GalpA-(1→ (residue B) are the principal structural units of DTP. Because the ring resonances of the two residues overlapped, they are collectively designated residue A/B and are presented as a single entry in Table 3. In the HSQC spectrum (Figure 1D), the anomeric H1/C1 signal of residues A/B was found at 4.84/100.36 ppm. From this anomeric proton, sequential correlations of H2/C2 (3.57/67.71 ppm), H3/C3 (3.86/67.66 ppm), H4/C4 (4.33/78.85 ppm), and H5/C5 (4.96/70.36 ppm) were established with the aid of the COSY (Figure 1E) and TOCSY (Figure 1F) spectra, enabling complete assignment of the dominant GalA residues of the HG backbone (Table 3). Several weak cross-peaks in the anomeric region (e.g., 104.29/4.50 ppm and 107.31/4.96 ppm) were tentatively attributed to minor residues such as galactose and arabinose; however, their low abundance and serious signal overlap precluded unambiguous assignment.

Taken together, DTP is characterized as a pectic polysaccharide that is mainly composed of a linear, partially methyl-esterified HG backbone of 1,4-linked α-GalpA residues. Although the detection of Rha and neutral sugar side chains implies the existence of RG-I domains, direct NMR evidence for the HG-RG-I linkage was not obtained, probably because of the low abundance of RG-I regions and the overlap of their signals.

### 3.2. DTP Ameliorates HFD-Induced Adipose Accumulation and Related Physiological Changes

As shown in Figure 2, an obese phenotype was successfully established after eight weeks of dietary intervention. The final body weight of the Model group reached 30.37 ± 1.72 g, which was significantly higher than the 26.37 ± 1.33 g observed in the Control group (*p* < 0.01). Accordingly, the body weight gain in the Model group reached 5.58 ± 1.12 g, whereas the Control group gained only 3.52 ± 0.42 g. DTP intervention effectively curbed this trend. Notably, the DTP-M group showed the most pronounced effect, with a final body weight of 28.30 ± 1.73 g and a weight gain of 3.60 ± 0.79 g, representing a reduction in weight gain of approximately 35.5% compared to the Model group (*p* < 0.01). Furthermore, while the daily energy intake in the Model group (17.92 ± 2.36 kcal/day) was markedly higher than in the Control group (12.71 ± 1.41 kcal/day), both medium and high doses of DTP significantly reduced energy intake to 15.16 ± 2.06 and 15.44 ± 1.55 kcal/day, respectively (*p* < 0.05).

The HFD-induced obesity was accompanied by increased organ and adipose tissue indices (Figure 2). The Model group displayed a significantly elevated liver index of 4.78 ± 0.71% compared to 4.12 ± 0.23% in the Control group. Intervention with DTP-M and DTP-H significantly lowered the liver index to 4.26 ± 0.42% and 4.26 ± 0.24%, respectively (*p* < 0.05). More critically, DTP targeted visceral fat accumulation. The eWAT and iWAT indices in the Model group surged to 2.79 ± 0.50% and 2.10 ± 0.41%, respectively. In contrast, DTP-M treatment dramatically reduced these indices to 1.65 ± 0.32% and 1.04 ± 0.20% (*p* < 0.01), levels that were much closer to the Control group. Similarly, sWAT index was reduced from 1.27 ± 0.36% in the Model group to 0.82 ± 0.30% in the DTP-M group (*p* < 0.05). No significant differences were observed in the iBAT index among groups.

### 3.3. DTP Ameliorates Glucose Metabolic Disorders in HFD-Fed Mice

HFD feeding caused severe disruption of glucose homeostasis, as illustrated in Figure 3. The fasting blood glucose (FBG) of the Model group rose to 8.50 ± 1.16 mmol/L, significantly higher than the 5.20 ± 0.69 mmol/L of the Control group (*p* < 0.01). DTP intervention reversed this hyperglycemia in a dose-dependent manner. Specifically, the FBG levels in the DTP-M and DTP-H groups decreased to 4.90 ± 0.46 mmol/L and 4.80 ± 0.55 mmol/L, respectively, showing no statistical difference from the Control group. Consistently, serum FINS levels were elevated to 28.79 ± 4.89 mU/L in the Model group but were significantly downregulated to 21.18 ± 1.66 mU/L following DTP-M treatment (*p* < 0.05). Consequently, the HOMA-IR index, a marker of insulin resistance, was markedly reduced from 10.68 ± 1.45 in the Model group to 3.96 ± 0.74 in the DTP-H group (*p* < 0.01).

During the OGTT (Figure 3D), the Model group exhibited sustained hyperglycemia, with an AUC value of 1604.30 ± 185.55, which was significantly higher than that of the Control group (1259.50 ± 98.95, *p* < 0.01). All DTP-treated groups exhibited improved glucose tolerance, with the AUC in the DTP-H group dropping to 1242.50 ± 81.32, effectively restoring it to normal levels. Additionally, liver glycogen content, which was depleted to 5.74 ± 3.29 mg/g in the Model group, was significantly replenished by DTP treatment, reaching 13.50 ± 4.02 mg/g in the DTP-H group (*p* < 0.05).

### 3.4. DTP Ameliorates HFD-Induced Dyslipidemia in HFD-Fed Mice

As shown in Figure 4, serum TC, TG, and LDL-C levels in the Model group were significantly elevated to 5.06 ± 0.23, 1.26 ± 0.14, and 0.88 ± 0.09 mmol/L, respectively, compared to the Control group (*p* < 0.01). DTP administration significantly mitigated these abnormalities. For instance, DTP-M treatment reduced serum TC by approximately 11.5% to 4.48 ± 0.37 mmol/L (*p* < 0.01) and lowered LDL-C to 0.63 ± 0.13 mmol/L (*p* < 0.05).

Regarding hepatic steatosis (Figure 4), the liver TC and TG contents in the Model group were 0.047 ± 0.005 and 0.103 ± 0.007 mmol/gprot, respectively, indicating severe lipid accumulation compared to the Control group (*p* < 0.01). DTP intervention effectively blocked this lipid deposition. In the DTP-H group, hepatic TC and TG levels were significantly decreased to 0.026 ± 0.006 and 0.038 ± 0.017 mmol/gprot, respectively (*p* < 0.01), indicating a protective effect against fatty liver.

### 3.5. DTP Ameliorates Liver Oxidative Stress and Inflammation in HFD-Fed Mice

HFD feeding triggered oxidative stress in the liver (Figure 5). The liver MDA content in the Model group increased to 0.75 ± 0.11 nmol/mgprot, whereas the activities of SOD, GSH-Px, and CAT were suppressed to 83.07 ± 12.11, 237.34 ± 51.14, and 36.72 ± 2.82 U/mgprot, respectively (*p* < 0.01). DTP treatment restored the antioxidant defense system. In the DTP-H group, MDA levels were reduced to 0.25 ± 0.03 nmol/mgprot, and SOD activity was restored to 117.85 ± 10.78 U/mgprot, comparable to the Control group.

Furthermore, inflammation was assessed by measuring cytokine levels (Figure 5). In the Model group, serum levels of pro-inflammatory cytokines IL-1β, IL-6, and TNF-α spiked to 123.56 ± 8.32, 129.51 ± 7.63, and 481.02 ± 68.84 pg/mL, respectively. Conversely, the anti-inflammatory cytokine IL-10 dropped to 123.08 ± 28.02 pg/mL. DTP intervention effectively rebalanced this inflammatory profile. Specifically, DTP-H significantly lowered serum TNF-α to 284.68 ± 23.90 pg/mL and elevated IL-10 to 217.41 ± 16.59 pg/mL (*p* < 0.01). A similar trend was observed in liver tissues, where DTP-M reduced hepatic IL-6 levels from 48.20 ± 7.46 in the Model group to 32.94 ± 4.82 pg/mgprot (*p* < 0.05).

### 3.6. DTP Modulates the Gut Microbiota in HFD-Fed Mice

#### 3.6.1. DTP Ameliorates HFD-Induced Dysbiosis by Improving Alpha and Beta Diversity

Alpha and beta diversity analyses were conducted to evaluate the effects of HFD and DTP on the gut microbial community structure. The coverage index of all samples exceeded 99.8% (Figure 6A–F), confirming adequate sequencing depth for capturing the microbial diversity. Regarding alpha diversity, no significant differences in community richness (Ace and Chao indices) or diversity (Shannon index) were found between the Control and Model groups (*p* > 0.05), whereas the Simpson index was markedly higher in the Model group than in the Control group (*p* < 0.05). DTP administration did not significantly alter the Shannon, Ace, or Chao indices relative to the Model group; however, the Simpson index of the DTP-H group was significantly lower than that of the Model group (*p* < 0.05) and approached the level of the Control group.

Principal coordinates analysis (PCoA) and non-metric multidimensional scaling (NMDS) based on beta diversity showed a distinct separation between the Control and Model groups (Figure 6G,H), demonstrating that HFD feeding substantially reshaped the overall structure of the gut microbiota.

#### 3.6.2. DTP Regulates the Composition of Gut Microbiota in HFD-Fed Mice

As shown in Figure 7A, HFD feeding induced significant shifts in the gut microbial composition at the phylum level. Compared with the Control group, the Model group showed a significantly increased relative abundance of Firmicutes and a decreased abundance of Bacteroidota (*p* < 0.01), leading to a markedly elevated F/B ratio. DTP intervention did not significantly reverse the F/B ratio at the phylum level. However, a more in-depth analysis at the genus level revealed that DTP exerted a precise restructuring effect on specific metabolic-related taxa.

At the genus level (Supplementary Table S1), the Model group displayed significantly lower relative abundances of *norank_f_Muribaculaceae*, *Bifidobacterium*, *Lachnospiraceae NK4A136 group* and *Enterorhabdus* (*p* < 0.01) compared with the Control group. In contrast, the relative abundances of genera linked to metabolic dysfunction, including *Allobaculum*, *Ileibacterium*, *Faecalibaculum*, and *Dubosiella*, were significantly higher (*p* < 0.05). DTP intervention selectively modulated these HFD-induced alterations through dose-dependent patterns. The DTP-M group exhibited a potent suppressive effect on HFD-enriched genera, significantly reducing the relative abundances of *Allobaculum*, *Ileibacterium*, and *Dubosiella* (*p* < 0.05) compared to the Model group. Although *Bifidobacterium* in the DTP-M group was not significantly elevated, the DTP-H group significantly increased the relative abundance of *Bifidobacterium* relative to the Model group (*p* < 0.01), restoring it to a level statistically comparable to that of the Control group.

To further pinpoint the key bacterial taxa that statistically differentiated the experimental groups, Linear discriminant analysis Effect Size (LEfSe) was employed to identify significant biomarkers (LDA score > 4.0, *p* < 0.05, Figure 7B,C). The analysis revealed that the predominant biomarkers for the Control group were from the phylum Bacteroidota, including the family Muribaculaceae and the genus *norank_f_Muribaculaceae*. In contrast, the key biomarkers for the Model group were from the phylum Firmicutes, including the family Erysipelotrichaceae and the genera *Allobaculum*, *Ileibacterium*, and *Dubosiella*. Following the intervention, the DTP-L group was significantly enriched with *Candidatus Saccharimonas* from the phylum Patescibacteria. The DTP-M group was characterized by an enrichment of the family Lachnospiraceae and the genus *Lachnospiraceae NK4A136 group* (phylum Firmicutes). Notably, the DTP-H group was enriched with the family Bifidobacteriaceae and the genus *Bifidobacterium* (phylum Actinobacteriota), as well as the genera *Faecalibaculum* and *Lachnoclostridium* (phylum Firmicutes). These findings reinforce the relative abundance data, indicating that DTP administration leads to a significant and targeted shift in the gut microbial community by modulating specific taxa.

### 3.7. Correlation Analysis Between Gut Microbiota and Metabolic Parameters

To further explore the potential links between the gut ecosystem and host physiology beyond group-level comparisons, Spearman’s correlation analysis was performed to examine whether the bacterial taxa significantly regulated by DTP were associated with the observed improvements in glucolipid metabolism.

At the phylum level (Figure 7D), the analysis showed established patterns of dysbiosis. The relative abundance of Firmicutes showed significant positive correlations with pro-inflammatory cytokines and adverse lipid profiles, while being negatively correlated with the anti-inflammatory cytokine IL-10 and the antioxidant enzyme CAT (*p* < 0.05). Conversely, Bacteroidota and Actinobacteriota displayed opposing correlation patterns (*p* < 0.05), supporting their general roles in maintaining metabolic homeostasis.

The analysis at the genus level provided more targeted insights, further supporting the functional importance of the specific genera modulated by our intervention (Figure 7E). Notably, the abundance of *Bifidobacterium*, which was significantly increased by high-dose DTP, demonstrated a robust positive correlation with beneficial markers such as liver glycogen, serum ADPN and anti-inflammatory IL-10 (*p* < 0.01). Furthermore, it was significantly and negatively correlated with a suite of detrimental parameters, including hepatic TG, the AUC of the OGTT, serum LDL-C, serum TG, and pro-inflammatory cytokines (*p* < 0.05). This pattern suggests that the promotion of *Bifidobacterium* may be an important contributor to the beneficial effects of DTP. Similarly, *norank_f_Muribaculaceae*, a genus suppressed by the HFD, was positively linked to antioxidant and anti-inflammatory markers. In stark contrast, genera whose proliferation was inhibited by DTP, such as *Allobaculum*, *Ileibacterium*, and *Dubosiella*, exhibited significant positive correlations with multiple adverse metabolic parameters, including hepatic TG, MDA, and FBG (*p* < 0.05). This opposing correlation pattern between beneficial and detrimental bacteria, whose abundances were directly influenced by DTP, highlights their association with the metabolic outcomes.

In summary, the correlation analysis indicated that the specific microbes (*Bifidobacterium*, *Allobaculum*) that were significantly modulated by DTP were also the taxa most closely correlated with improved host metabolic health. This suggests that the ameliorative effects of DTP on glucolipid metabolic disorders may be partially linked to the selective regulation of these key functional players in the gut ecosystem.

## 4. Discussion

In this work, DTP was structurally characterized as an acidic pectic polysaccharide with an HG-dominated backbone, a molecular weight of 52 kDa, and a high GalA content (72.7%), which is consistent with the typical features of pectins that participate in metabolic regulation [36]. It is worth noting that the DM of DTP was only 34.12%, classifying it as a low-methoxyl pectin (LMP). LMPs have been reported to form gels with calcium ions in the intestinal tract, thereby entrapping lipids and delaying gastric emptying [36,37]. Such structural properties presumably constitute the material basis underlying the metabolic benefits of DTP observed in our animal experiments.

Consistent with the reported bioactivities of pectic polysaccharides, DTP administration alleviated a series of HFD-induced metabolic abnormalities in the present study, covering obesity, insulin resistance, and dyslipidemia. These benefits, including the inhibition of body weight gain, the reduction of visceral fat, and the amelioration of hepatic steatosis, are in agreement with the currently accepted mechanisms of dietary pectins, namely delayed gastric emptying, bile acid binding and cholesterol excretion, and modulation of gut microbial metabolism [37]. Among the three doses, DTP-M produced the strongest anti-obesity effect, reducing body weight gain by 35.5% relative to the Model group, together with a notable decline in daily energy intake (from 17.92 to 15.16 kcal/day), which implies that DTP may promote satiety through the viscosity of its HG-rich structure. DTP also preferentially reduced visceral adiposity, as reflected by the 40.8% and 50.4% decreases in eWAT and iWAT indices, respectively, in the DTP-M group. With respect to lipid metabolism, serum TC and LDL-C elevations induced by the HFD were markedly reversed by DTP, whereas HDL-C remained elevated, which is regarded as a typical adaptive response to dietary cholesterol in C57BL/6J mice [38]. More importantly, hepatic lipid accumulation was largely prevented, with hepatic TC and TG decreasing by more than 50% in the DTP-H group. Collectively, these observations suggest that DTP restricts energy intake and, meanwhile, regulates lipid clearance and storage pathways.

The improvements in glucose homeostasis were equally notable. DTP administration lowered fasting blood glucose and insulin levels, ameliorated insulin resistance (lower HOMA-IR), and restored hepatic glycogen storage. In the DTP-H group, fasting blood glucose returned to a level close to that of the Control group (4.80 mmol/L), and the HOMA-IR index declined substantially from 10.68 to 3.96, indicating improved insulin sensitivity. Of particular interest is the recovery of liver glycogen: the glycogen content depleted by approximately 57% in the Model group was almost fully restored by high-dose DTP. Because hepatic glycogen synthesis is directly regulated by insulin signaling, this recovery implies an amelioration of hepatic insulin resistance, which is probably associated with the attenuation of hepatic oxidative stress and inflammation. Indeed, DTP-H normalized hepatic SOD activity, decreased MDA content by 66.7%, downregulated the pro-inflammatory cytokines TNF-α and IL-6, and upregulated the anti-inflammatory cytokine IL-10 in both serum and liver, thus creating an anti-inflammatory microenvironment favorable for metabolic recovery.

The gut microbiota is a central regulator of host metabolism, and its dysbiosis is deeply involved in the pathogenesis of HFD-induced metabolic disorders [39]. In this study, the HFD caused a typical dysbiosis with increased Firmicutes and decreased Bacteroidota; however, DTP did not simply restore the F/B ratio at the phylum level but rather reshaped the community in a dose-dependent manner at the genus level. The DTP-M group, which exhibited the most pronounced weight loss, was characterized by the specific suppression of *Allobaculum* (from 18.53% in Model to 2.33%). *Allobaculum* (family Erysipelotrichaceae) has been recognized as an important promoter of obesity and is positively associated with ileal inflammation [40]. In line with this, our correlation analysis showed that *Allobaculum* abundance was positively correlated with body weight, hepatic TG, and serum inflammatory cytokines, suggesting that reducing this pathobiont may contribute to the observed weight control.

Conversely, the DTP-H group exhibited a marked prebiotic effect, increasing the abundance of *Bifidobacterium* to 4.94%, which was comparable to that of the Control group (3.84%). From a mechanistic point of view, as a low-methoxyl pectic polysaccharide dominated by a linear HG backbone, DTP cannot be readily hydrolyzed by host digestive enzymes in the upper gastrointestinal tract [36] and therefore reaches the colon largely intact. Its structural characteristics, namely the low degree of methyl-esterification and the presence of neutral sugar side chains (e.g., RG-I domains), make DTP an ideal selective substrate for certain beneficial commensals, particularly *Bifidobacterium* species, which harbor abundant carbohydrate-active enzymes (CAZymes) and polysaccharide utilization loci (PULs) dedicated to degrading complex pectic structures [41]. This targeted enzymatic degradation reasonably accounts for the significant enrichment of *Bifidobacterium* after high-dose DTP administration [42]. The increased abundance of *Bifidobacterium* was positively correlated with beneficial outcomes, including higher liver glycogen and serum IL-10 levels (*p* < 0.01), in agreement with the established roles of *Bifidobacterium* in maintaining intestinal barrier integrity, modulating host immunity, and ameliorating insulin resistance [43]. Altogether, these results suggest that DTP functions in a “dual-target” manner, i.e., suppressing HFD-driven opportunistic pathogens (such as *Allobaculum*) at the medium dose while promoting beneficial commensals (such as *Bifidobacterium*) at the high dose, which may explain the dose-specific phenotypic improvements, namely the greater weight loss in DTP-M and the maximal insulin sensitization in DTP-H.

Several limitations of this study should be acknowledged. First, although 16S rRNA gene sequencing and Spearman’s correlation analysis revealed significant associations between the DTP-modulated taxa (e.g., *Bifidobacterium* and *Allobaculum*) and host metabolic parameters, such associative evidence cannot establish a causal relationship between gut microbiota remodeling and the metabolic benefits of DTP. Further studies employing fecal microbiota transplantation or antibiotic-induced microbiota depletion are warranted to verify the causal role of the gut microbiota. Second, the taxonomic and functional resolution of 16S rRNA gene sequencing is limited; metagenomic sequencing and metabolite profiling (e.g., short-chain fatty acids) would provide deeper mechanistic insights. Third, a DTP-only control group was not included in the present design, and the potential effects of DTP on normal mice remain to be investigated in the future. Finally, only the HFD-fed mice with the greatest body weight gain (top 60%) were included in the intervention experiment; although this prespecified criterion ensured a uniform obese phenotype, it may limit the generalizability of the findings to the full spectrum of HFD-induced responses, and the potential selection bias should be taken into account when interpreting the results.

## 5. Conclusions

In conclusion, a novel acidic pectic polysaccharide, DTP, mainly consisting of an *α*-1,4-linked homogalacturonan backbone with 34.12% methyl-esterification, was obtained from Dancong tea in this study. DTP administration markedly alleviated HFD-induced obesity, insulin resistance, dyslipidemia, and hepatic steatosis in mice. These beneficial effects were accompanied by the attenuation of oxidative stress and inflammation, as well as a specific remodeling of the gut microbiota composition, in particular the suppression of opportunistic genera such as *Allobaculum* and the enrichment of beneficial *Bifidobacterium*. Correlation analysis indicated that these microbial shifts were closely associated with the improved metabolic parameters. These findings highlight the potential of DTP as a promising functional food ingredient for the management of metabolic diseases, although the causal role of gut microbiota modulation in mediating these benefits warrants further investigation.

## Figures and Tables

**Figure 1:**
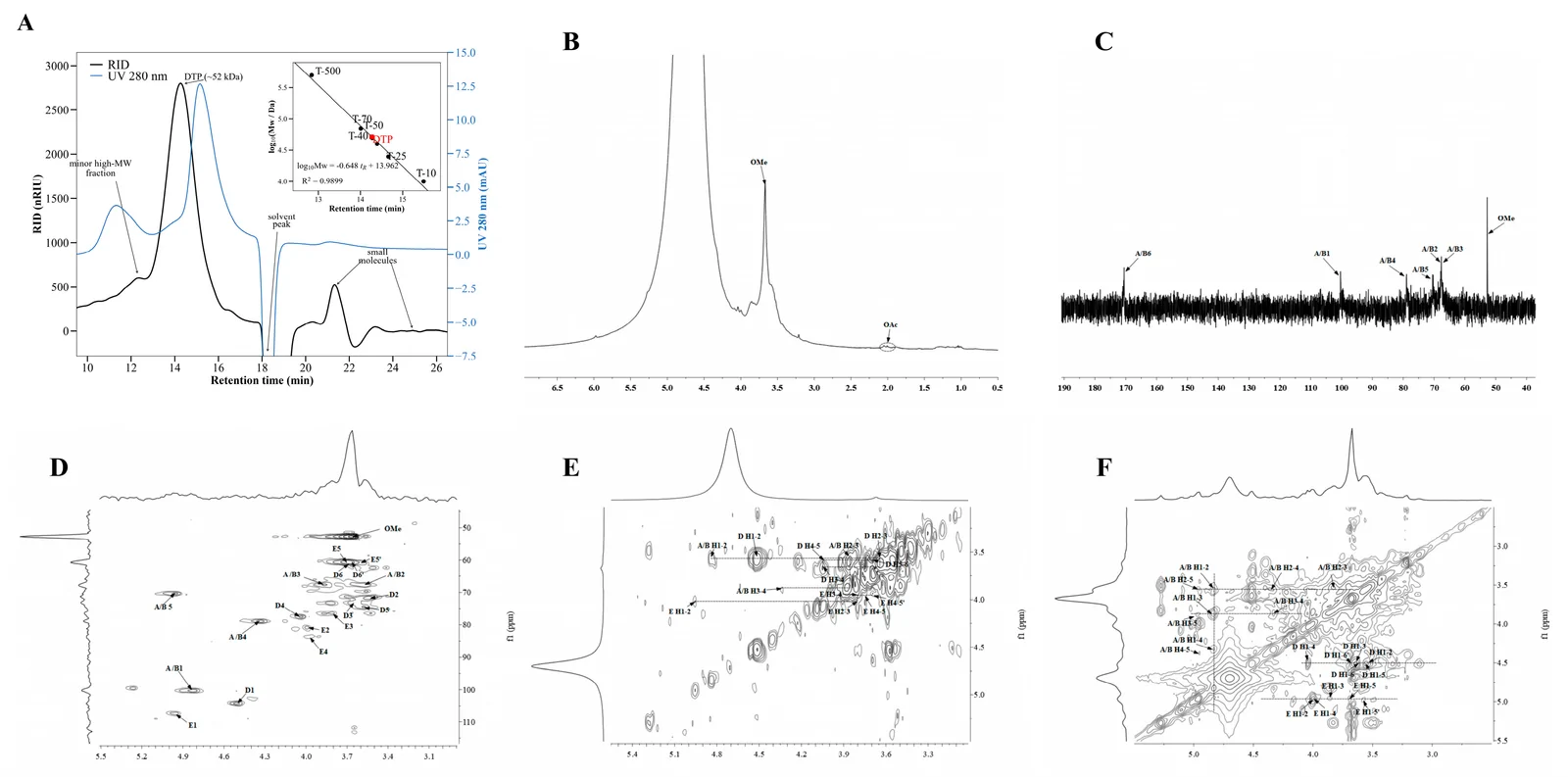
Structural characterization of DTP. (**A**) HPGPC chromatogram of DTP with the dextran calibration curve (inset) and designation of the chromatographic components; (**B**) ^1^H NMR spectrum; (**C**) ^13^C NMR spectrum; (**D**) HSQC spectrum; (**E**) COSY spectrum; (**F**) TOCSY spectrum. The sharp negative deflection at approximately 18.5 min (with a concurrent deflection in the UV trace) is the solvent system peak at the total permeation volume of the column and does not correspond to any sample component.

**Figure 2:**
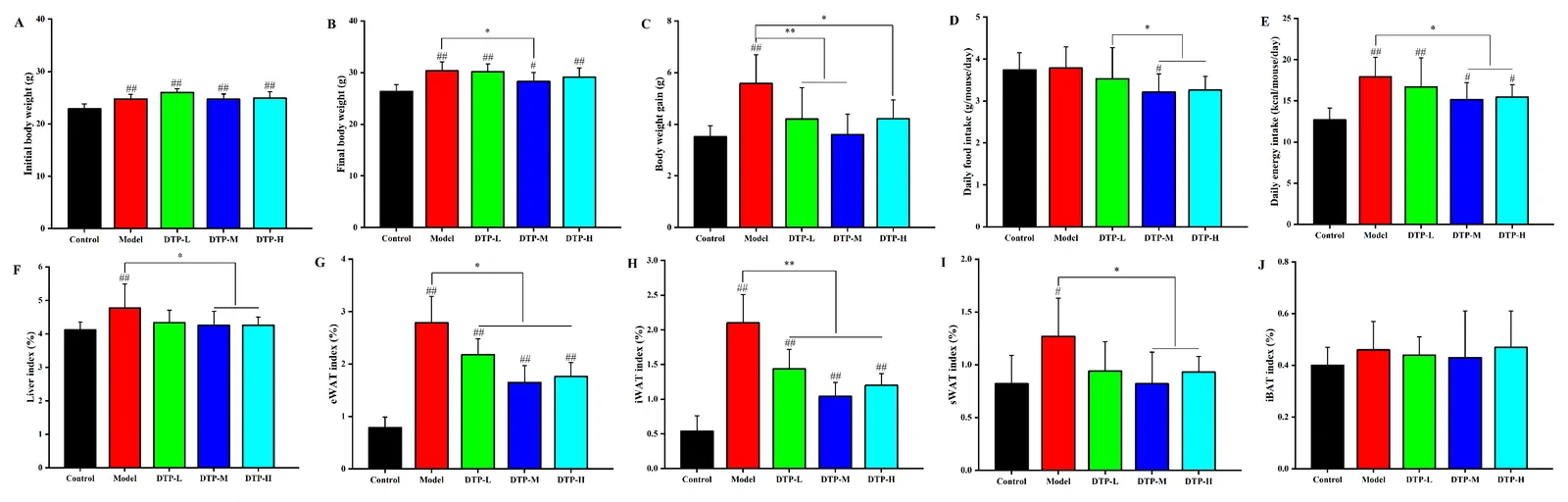
Effects of DTP on body weight, energy intake, and tissue indices in HFD-fed mice. (**A**) Initial body weight; (**B**) Final body weight; (**C**) Body weight gain; (**D**) Daily food intake; (**E**) Daily energy intake; (**F**) Liver index; (**G**) eWAT index; (**H**) iWAT index; (**I**) sWAT index; (**J**) iBAT index. # *p* < 0.05, ## *p* < 0.01 vs. Control group; * *p* < 0.05, ** *p* < 0.01 vs. Model group.

**Figure 3:**
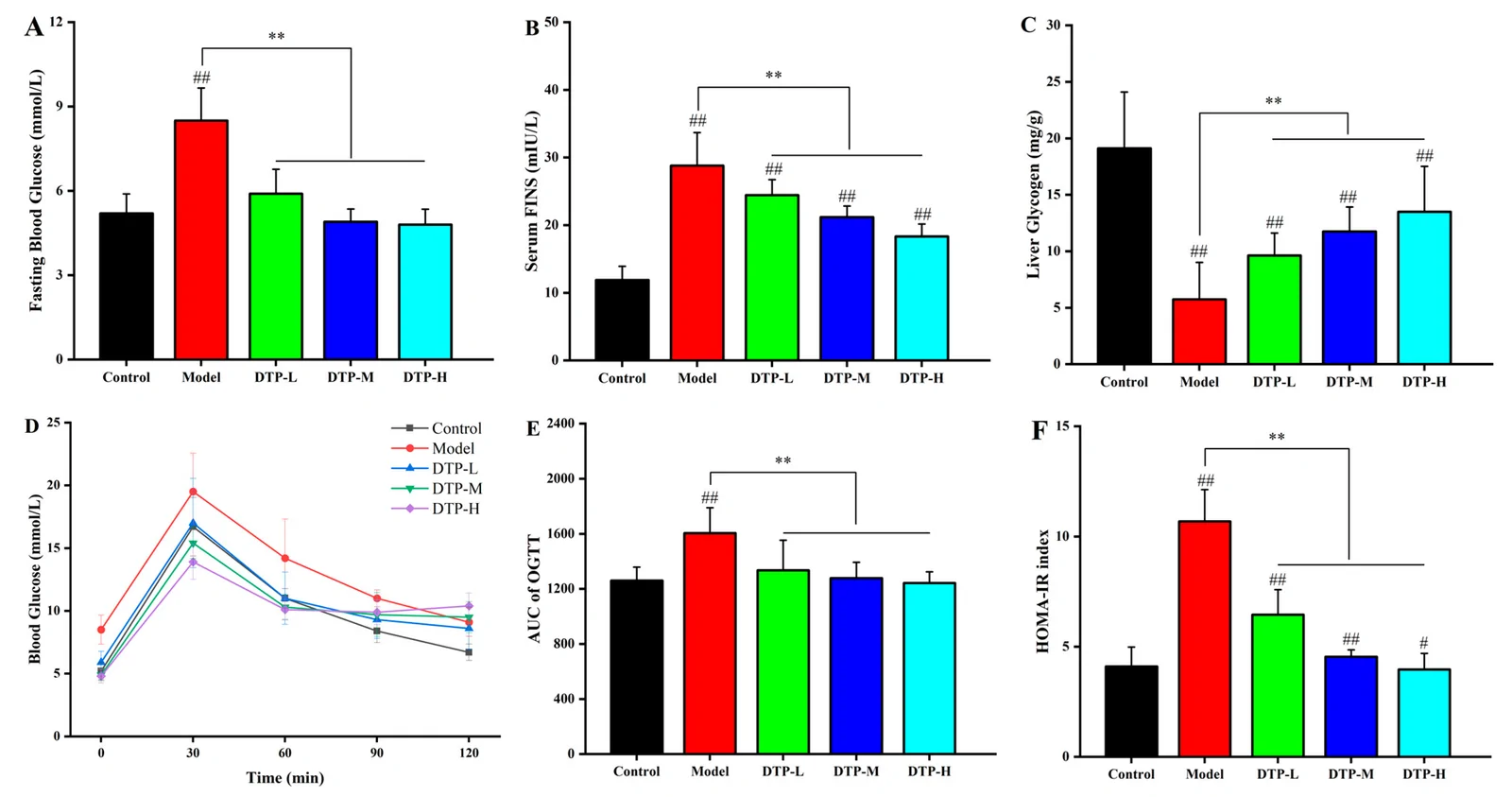
Effects of DTP on glucose metabolism in HFD-fed mice. (**A**) FBG; (**B**) Serum insulin; (**C**) Liver glycogen; (**D**) OGTT; (**E**) AUC for the OGTT; (**F**) HOMA-IR index. # *p* < 0.05, ## *p* < 0.01 vs. Control group; ** *p* < 0.01 vs. Model group.

**Figure 4:**
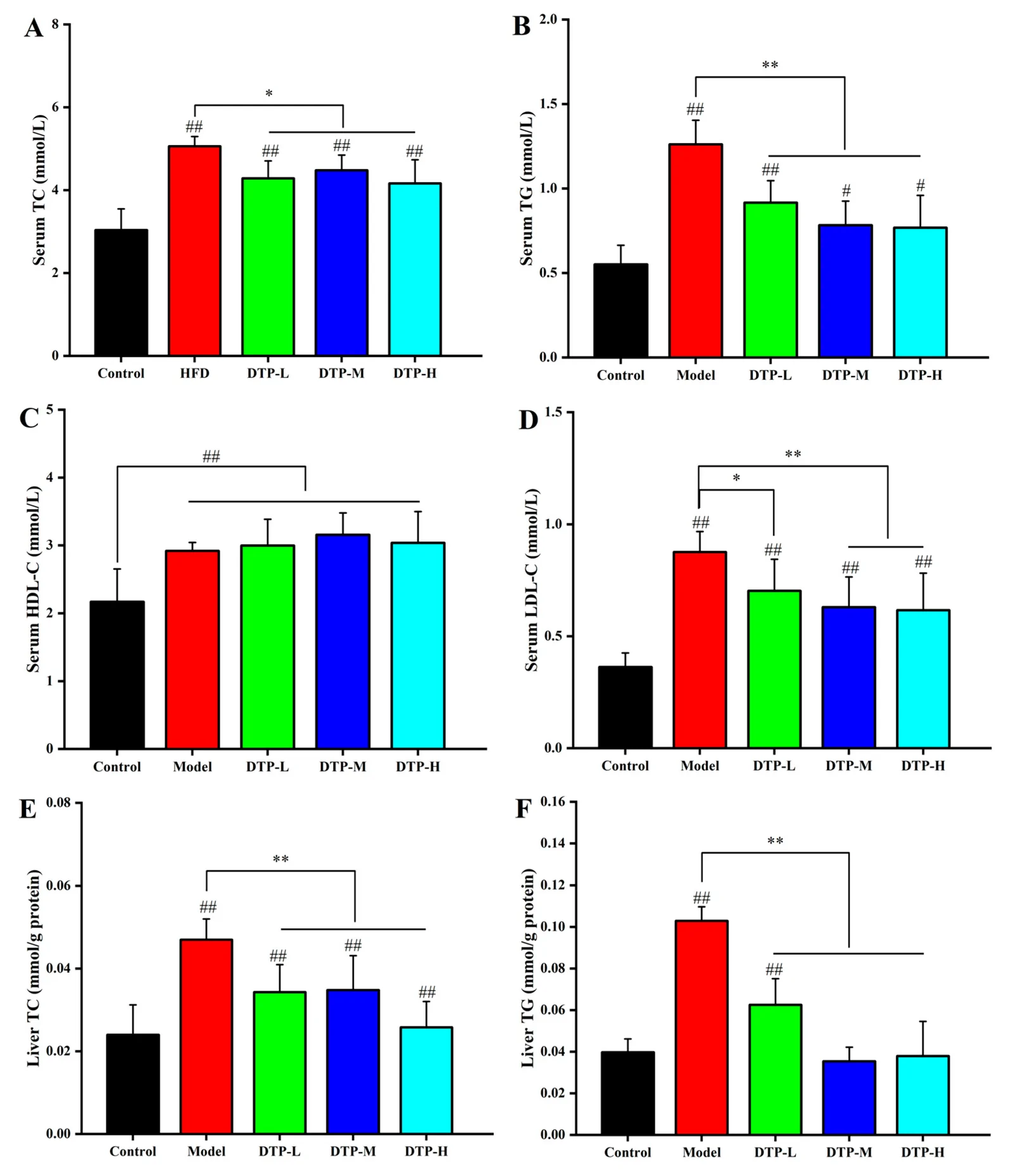
Effect of DTP on lipid profiles in serum and liver of HFD-fed mice. (**A**) Serum TC; (**B**) Serum TG; (**C**) Serum HDL-C; (**D**) Serum LDL-C; (**E**) Liver TC; (**F**) Liver TG. # *p* < 0.05, ## *p* < 0.01 vs. Control group; * *p* < 0.05, ** *p* < 0.01 vs. Model group.

**Figure 5:**
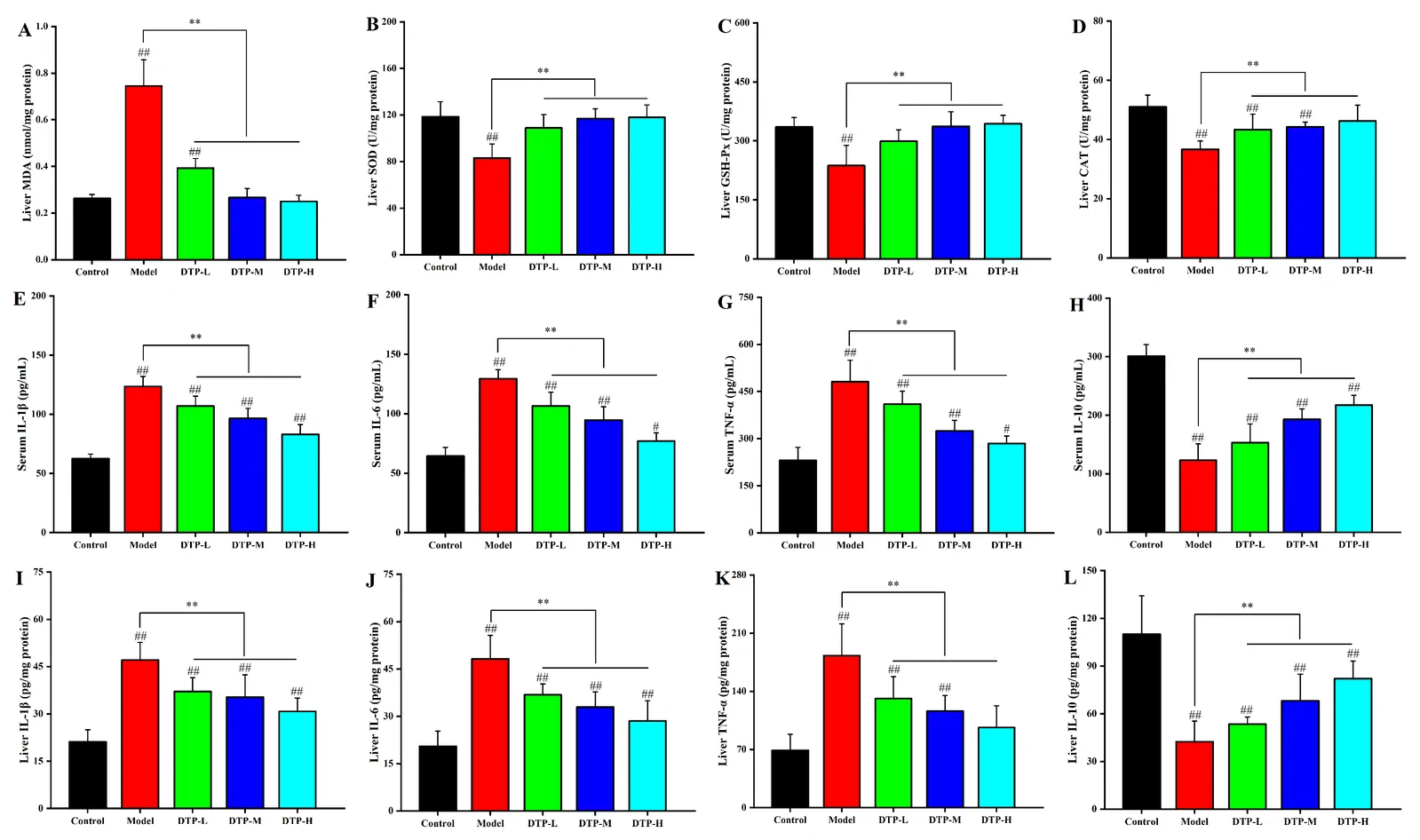
Effects of DTP on hepatic oxidative stress and inflammatory responses in HFD-fed mice. (**A**) Liver MDA content; (**B**) Liver SOD activity; (**C**) Liver GSH-Px activity; (**D**) Liver CAT activity; (**E**) Serum IL-1β levels; (**F**) Serum IL-6 levels; (**G**) Serum TNF-α levels; (**H**) Serum IL-10 levels; (**I**) Liver IL-1β levels; (**J**) Liver IL-6 levels; (**K**) Liver TNF-α levels; (**L**) Liver IL-10 levels. # *p* < 0.05, ## *p* < 0.01 vs. Control group; ** *p* < 0.01 vs. Model group.

**Figure 6:**
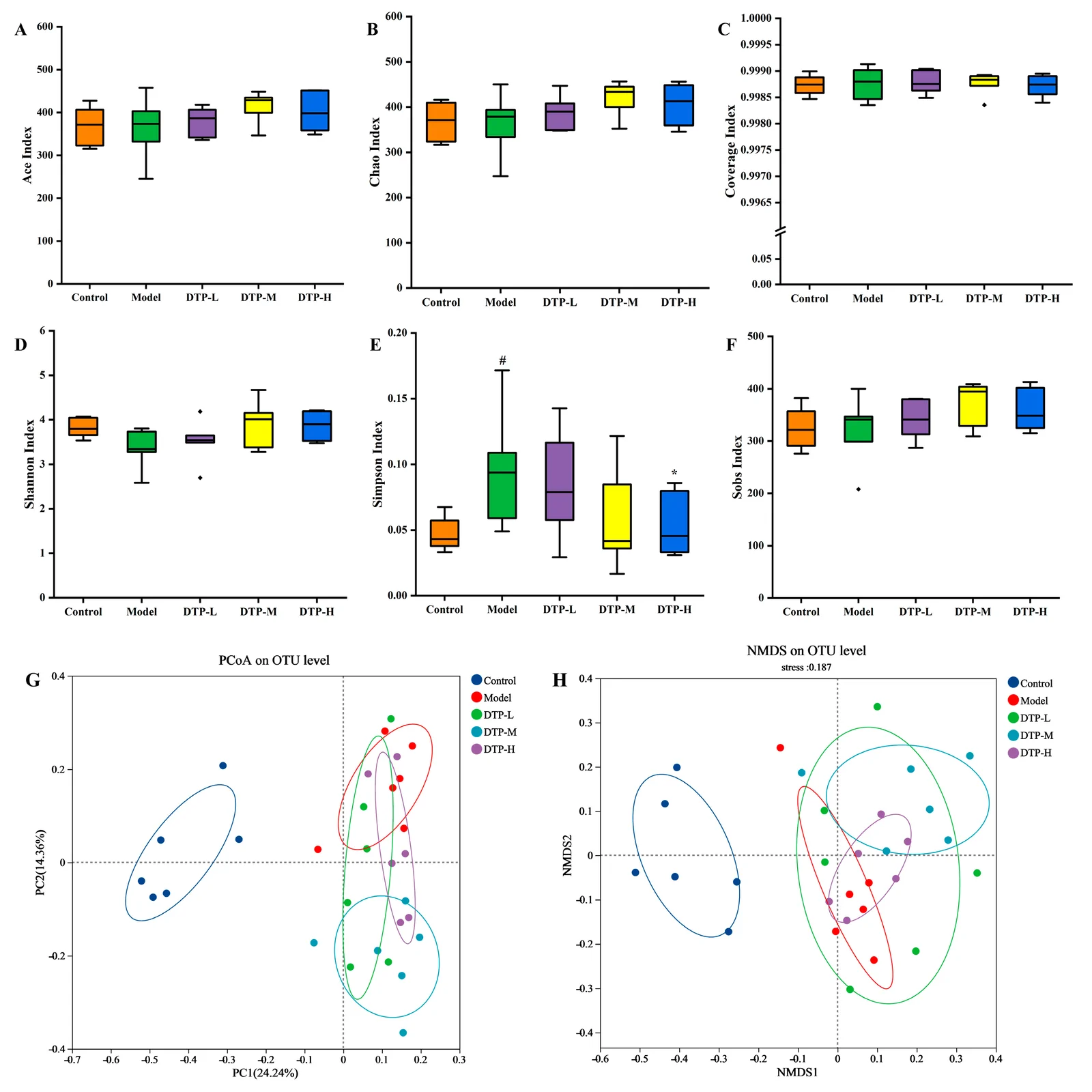
DTP modulates the gut microbiota diversity in HFD-fed mice. (**A**–**F**) Alpha diversity (including Ace, Chao, Shannon, and Simpson indices). (**G**) Principal Coordinates Analysis (PCoA) plot and (**H**) Non-metric Multidimensional Scaling (NMDS) plot based on beta diversity. Data are presented as mean ± SEM (*n* = 6). # *p* < 0.05 vs. Control group; * *p* < 0.05 vs. Model group; ◆ represents outlier data points.

**Figure 7:**
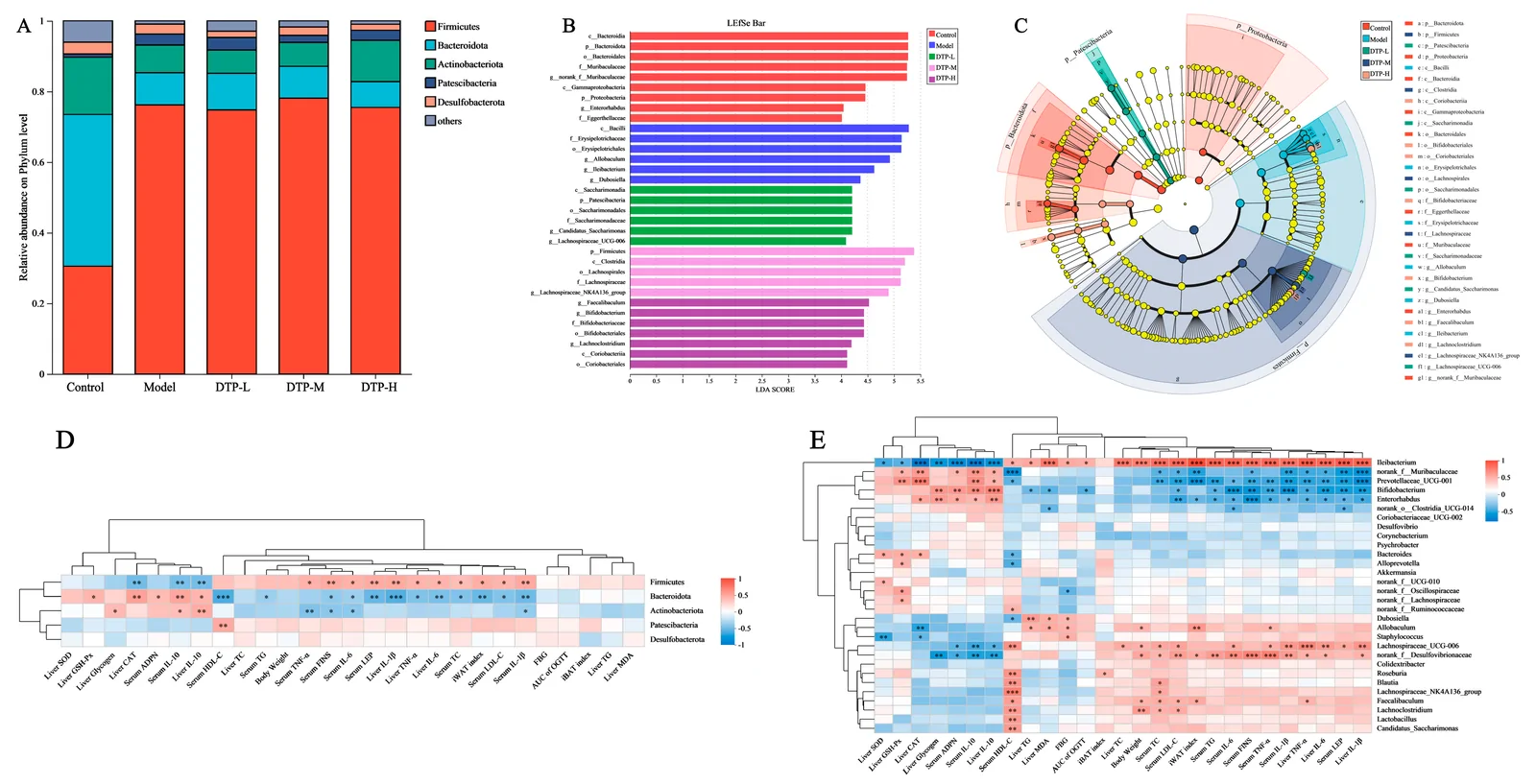
DTP regulates the gut microbiota composition and its association with host metabolic parameters in HFD-fed mice. (**A**) Relative abundance of gut microbiota at the phylum level. (**B**) Linear discriminant analysis (LDA) scores (LDA score > 4.0). (**C**) LEfSe cladogram identifying significant microbial biomarkers among groups. (**D**) Spearman’s correlation heatmap between gut microbiota at the phylum level and parameters related to glucolipid metabolism. (**E**) Spearman’s correlation heatmap between gut microbiota at the genus level and metabolic parameters. Data are presented as mean ± SEM (*n* = 6). * *p* < 0.05, ** *p* < 0.01, *** *p* < 0.001.

**Table 1:** Monosaccharide composition of DTP.

Monosaccharide	Molar Percentage (%)
Fucose (Fuc)	0.5 ± 0.0
Rhamnose (Rha)	5.4 ± 0.8
Arabinose (Ara)	2.0 ± 0.3
Galactose (Gal)	9.6 ± 0.3
Glucose (Glc)	6.1 ± 0.8
Xylose (Xyl)	0.8 ± 0.3
Mannose (Man)	1.7 ± 0.4
Ribose (Rib)	1.3 ± 0.1
Galacturonic acid (GalA)	72.7 ± 1.9
R_1_	4.3
R_2_	<0.1
R_3_	2.15

Data are presented as the mean ± SEM of three independent replicates (*n* = 3). The formulas used are as follows: R_1_ = GalA/(Rha + Ara + Gal); R_2_ = Rha/GalA; R_3_ = (Ara + Gal)/Rha.

**Table 2:** Glycosidic linkage analysis of DTP.

	PMAAs	Linkage Type	Percentage (%) ^a^
GalA	2,3,4,6-Me_4_-Gal	T-GalpA	1.55
	2,3,4,6-Me_4_-Gal	T-GalpAOMe	1.02
	2,3,6-Me_3_-Gal	1,4-GalpA	47.15
	2,3,6-Me_3_-Gal	1,4-GalpAOMe	24.42
	3,6-Me_2_-Gal	1,2,4-GalpA	0.92
	3,6-Me_2_-Gal	1,2,4-GalpAOMe	0.41
	2,3-Me_2_-Gal	1,4,6-GalpA	0.88
	2,3-Me_2_-Gal	1,4,6-GalpAOMe	0.75
Gal	2,3,4,6-Me_4_-Gal	T-Galp	0.95
	2,4,6-Me_3_-Gal	1,3-Galp	tr.
	2,3,6-Me_3_-Gal	1,4-Galp	3.14
	2,3,4-Me_3_-Gal	1,6-Galp	0.93
	2,6-Me_4_-Gal	1,3,4-Galp	2.69
	3,6-Me_2_-Gal	1,2,4-Galp	0.38
	2,3-Me_2_-Gal	1,4,6-Galp	0.32
	2,4-Me_2_-Gal	1,3,6-Galp	0.87
Ara	2,3,5-Me_3_-Ara	T-Araf	5.87
	2,3-Me_2_-Ara	1,5-Araf	1.17
Glc	2,3,6-Me_3_-Glc	1,4-Glcp	5.87
Rha	2,3,4-Me_3_-Rha	1,2-Rhap	0.77

tr., trace. ^a^ The percentage was calculated by peak area ratio.

**Table 3:** ^13^C and ^1^H chemical shifts of the major HG residues in DTP.

Residue	No.	C_1_/H_1_	C_2_/H_2_	C_3_/H_3_	C_4_/H_4_	C_5_/H_5_	C_6_/H_6_	OMe
→4)-*α*-GalpA-(→ and its C-6 methyl-esterified form (→4)-*α*-GalpA-6-OMe-(→)	A/B	100.36 4.84	67.71 3.57	67.66 3.86	78.85 4.33	70.36 4.96	170.60	52.74 3.67
→4)-α-GalpA-(→	B	100.36 4.84	67.71 3.57	67.66 3.86	78.85 4.33	70.36 4.96	170.60	

Note: the ring resonances of →4)-α-D-GalpA-(1→ (residue B) and its C-6 methyl-esterified form →4)-α-D-GalpA-6-OMe-(1→ (residue A) overlapped in the 1D 1H and 13C spectra, because C-6 methyl-esterification has only a minor influence on the chemical shifts of the GalpA ring carbons; the two residues are therefore presented as a single entry with shared resonances rather than as independently identified structural units. The methyl-esterified form was specifically evidenced by the diagnostic OMe signal at 52.74/3.67 ppm (13C/1H) and by the two-step carboxyl-reduction methylation analysis (Table 2).

## Data Availability

The data presented in this study are available on request from the corresponding author.

## Supplementary Materials

The following supporting information can be downloaded at: https://www.mdpi.com/article/10.3390/foods15193364/s1, Supplementary Table S1: Relative abundance (%) of the top 30 dominant bacterial genera in each group; Figure S1: HPGPC chromatograms (RID) of the polysaccharide before dialysis (WYJ-2) and after dialysis (DTP).

## Abbreviations

The following abbreviations are used in this manuscript:

ADPN	Adiponectin
ANOVA	Analysis of variance
AUC	Area under the curve
CAT	Catalase
CAZymes	Carbohydrate-active enzymes
COSY	Correlation spectroscopy
DB	Degree of branching
DM	Degree of methyl-esterification
DMSO	Dimethyl sulfoxide
DTP	Dancong tea polysaccharide
eWAT	Epididymal white adipose tissue
F/B	Firmicutes/Bacteroidota ratio
FBG	Fasting blood glucose
FINS	Fasting insulin
GalA	Galacturonic acid
GC-MS	Gas chromatography-mass spectrometry
GSH-Px	Glutathione peroxidase
HDL-C	High-density lipoprotein cholesterol
HFD	High-fat diet
HG	Homogalacturonan
HOMA-IR	Homeostatic model assessment for insulin resistance
HPAEC-PAD	High-performance anion exchange chromatography with pulsed amperometric detection
HPGPC	High-performance gel permeation chromatography
HSQC	Heteronuclear single quantum coherence
iBAT	Interscapular brown adipose tissue
IL	Interleukin
iWAT	Inguinal white adipose tissue
LDA	Linear discriminant analysis
LDL-C	Low-density lipoprotein cholesterol
LEfSe	Linear discriminant analysis effect size
LEP	Leptin
LPS	Lipopolysaccharide
MDA	Malondialdehyde
MWCO	Molecular weight cutoff
NAFLD	Non-alcoholic fatty liver disease
NMDS	Non-metric multidimensional scaling
NMR	Nuclear magnetic resonance
OGTT	Oral glucose tolerance test
PCoA	Principal coordinates analysis
PMAA	Partially methylated alditol acetate
PULs	Polysaccharide utilization loci
RG-I	Rhamnogalacturonan-I
SEM	Standard error of the mean
SOD	Superoxide dismutase
sWAT	Subcutaneous white adipose tissue
T2DM	Type 2 diabetes mellitus
TC	Total cholesterol
TFA	Trifluoroacetic acid
TG	Triglycerides
TNF-α	Tumor necrosis factor-α
TOCSY	Total correlation spectroscopy
