# Supplementary material for: Structural Features of a Dancong Tea Polysaccharide and Its Amelioration of Glucolipid Metabolic Disorders Associated with Gut Microbiota Modulation
Source: Foods. 2026 Sep 22;15(19):3364. doi: 10.3390/foods15193364 (PMC13664100; doi:10.3390/foods15193364)
Supplement: Supplementary file 1 [file foods-15-03364-s001.zip › foods-4521924-supplementary.pdf]

Supplementary Materials

Structural features of a Dancong tea polysaccharide and its amelioration of glucolipid metabolic disorders associated with gut microbiota modulation

Run Lin[a†], Zhe Wang[b†], Zhiyang Chen[a], Ruisen Cen[a], Junqiao Wang[b], Xiaoyong Wu[a, c, *]

[a] School of Food Science, Guangdong Pharmaceutical University, Zhongshan 528453, China;

[b] State Key Laboratory of Food Science and Resources, China-Canada Joint Lab of Food Science and Technology, Key Laboratory of Bioactive Polysaccharides of Jiangxi Province, Nanchang University, Nanchang 330047, China;

[c] Guangdong Engineering Technology Research Center of Medicine-Food Homologous Foods, Guangdong Pharmaceutical University, Zhongshan 528453, China

* Email: wuxiaoyong@gdpu.edu.cn (X. Wu)

[†] These authors have contributed equally to this work.

Supplementary Table S1. Relative abundance (%) of the top 30 dominant bacterial genera in each group. #*P*<0.05, ##*P*<0.01 vs. Control group; **P*<0.05, ***P*<0.01 vs. Model group

Species name	Control	Model	DTP-L	DTP-M	DTP-H
norank_f_Muribaculaceae	39.56±14.80	7.96±8.14##	7.01±4.79##	6.64±6.37##	5.70±2.79##
Lactobacillus	4.36±7.56	8.37±5.76	12.38±14.09	16.73±13.51#	11.29±2.49
Allobaculum	6.27±6.44	18.53±8.31#	8.18±8.69	2.33±1.30**	12.73±9.21
Lachnospiraceae NK4A136 group	1.33±1.00	4.70±1.95##	6.37±2.45##	16.65±9.69##*	8.92±7.28#
Staphylococcus	3.61±3.52	8.90±14.41	7.96±6.46	3.61±4.29	2.10±1.18
Corynebacterium	8.17±7.43	4.98±6.62	3.09±3.82	5.35±9.53	2.70±2.59
Ileibacterium	0.74±0.68	8.87±2.96##	6.65±7.05##	3.24±2.66*	3.34±2.19#
Faecalibaculum	0.56±0.76	2.96±1.31##	5.32±7.03#	4.49±3.85#	6.31±3.95##
Candidatu Saccharimonas	0.91±0.68	3.12±3.59	3.56±2.56#	1.98±1.30	2.84±0.82##
Bifidobacterium	3.84±2.88	0.47±0.19##	1.48±1.89#	0.44±0.53##	4.94±3.90**
Lachnoclostridium	0.53±0.21	1.72±1.33##	2.45±1.40##	2.73±1.31##	3.07±1.78##
Desulfovibrio	3.36±3.62	2.38±1.57	0.80±0.32#	1.64±1.05	1.32±1.03
Dubosiella	1.22±1.10	4.43±2.32#	0.19±0.14#**	0.70±0.75**	1.84±2.47
Lachnospiraceae_UCG-006	0.36±0.24	1.62±1.56#	2.46±1.80#	2.09±0.34##	1.11±0.64#
norank_o_Clostridia_UCG-014	2.72±2.97	1.01±0.39	0.80±0.66	0.76±0.44	1.61±0.80
norank_f_Lachnospiraceae	0.75±0.52	0.91±0.67	1.27±0.85	2.17±0.90#*	1.24±0.67
norank_f_Oscillospiraceae	0.53±0.34	0.57±0.39	1.10±1.33	1.75±1.37	2.13±2.47
Colidextribacter	0.28±0.15	1.04±1.03	0.75±0.92	2.73±3.08#	1.18±0.78#
Psychrobacter	4.09±6.33	0.27±0.39	0.18±0.25	1.22±2.62	0.03±0.04#
Coriobacteriaceae_UCG-002	0.93±0.98	1.16±1.17	0.37±0.48	0.40±0.64	2.12±0.95
Enterorhabdus	2.45±1.71	0.38±0.34##	0.60±0.47#	0.34±0.28#	0.40±0.16#
Bacteroides	0.54±0.46	0.33±0.37	1.07±1.41	0.68±0.99	0.63±0.49
Roseburia	0.12±0.09	0.34±0.45	0.34±0.17#	1.27±1.38#	1.17±1.35#
Blautia	0.07±0.06	0.47±0.68	0.16±0.19	1.87±2.17#	0.58±0.62#
norank_f_UCG-010	0.40±0.18	0.41±0.25	0.63±0.63	0.68±0.35	0.93±0.73
Akkermansia	0.18±0.27	0.48±0.64	1.35±2.91	0.24±0.49	0.61±0.66
Alloprevotella	0.51±0.66	0.39±0.65	1.10±2.56	0.34±0.37	0.45±0.55
norank_f_Desulfovibrionaceae	0.02±0.02	0.41±0.39##	0.99±1.22##	0.71±0.71##	0.39±0.48##
Prevotellaceae_UCG-001	1.86±3.63	0.03±0.04##	0.27±0.43	0.19±0.25	0.11±0.13#
norank_f_Ruminococcaceae	0.28±0.18	0.26±0.16	0.65±0.63	0.46±0.23	0.55±0.38

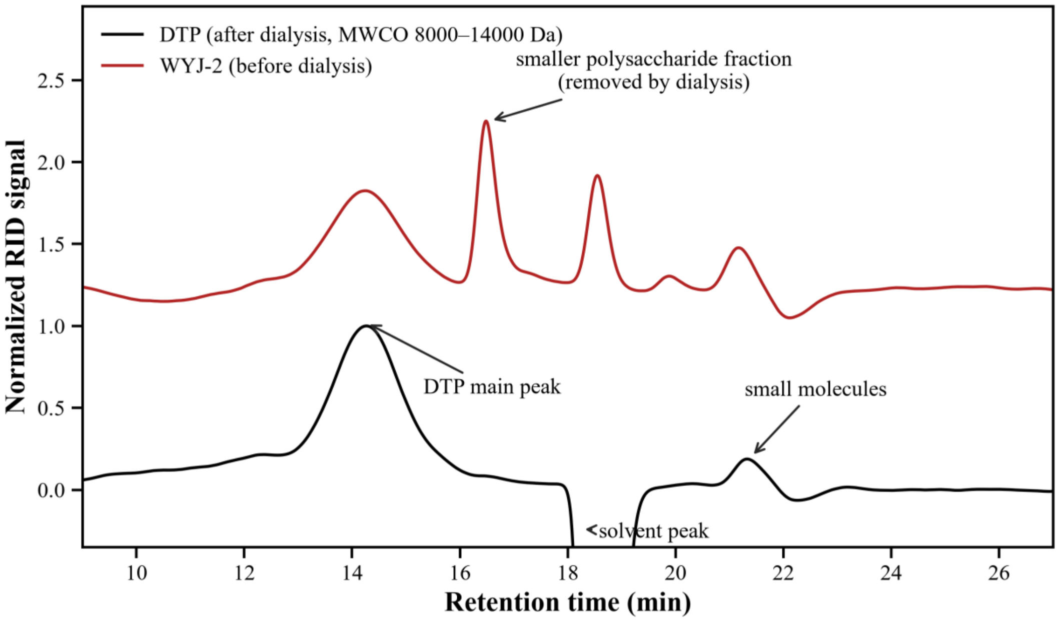

Figure S1. HPGPC chromatograms (RID) of the polysaccharide before dialysis (WYJ-2) and after dialysis (DTP). The smaller polysaccharide fraction eluting at approximately 16.5 min in the pre-dialysis sample was removed by the dialysis step (MWCO 8000–14000 Da); the small peaks remaining after the DTP main peak (approximately 20–23 min) correspond to small molecules rather than saccharide components. The sharp negative deflection at approximately 18.5 min is the solvent system peak.
